# Impact of Serum Glucose Levels on Outcomes in Acute Pancreatitis: A Retrospective Analysis

**DOI:** 10.3390/medicina60060856

**Published:** 2024-05-24

**Authors:** Marina Balaban, Daniel Vasile Balaban, Iulia Enache, Ioan Cristian Nedelcu, Mariana Jinga, Cristian Gheorghe

**Affiliations:** 1 Doctoral School, Carol Davila University of Medicine and Pharmacy, 020021 Bucharest, Romania; iulia.enache@drd.umfcd.ro (I.E.); ioan-cristian.nedelcu@drd.umfcd.ro (I.C.N.); 2 Internal Medicine and Gastroenterology Department, Carol Davila University of Medicine and Pharmacy, 020021 Bucharest, Romania; vasile.balaban@umfcd.ro (D.V.B.); mariana.jinga@umfcd.ro (M.J.); cristian.gheorghe@umfcd.ro (C.G.); 3 Gastroenterology Department, Central Military Emergency University Hospital, 010825 Bucharest, Romania; 4 Gastroenterology Department, Fundeni Clinical Institute, 022328 Bucharest, Romania

**Keywords:** acute pancreatitis, diabetes mellitus, on-admission serum glucose, peak serum glucose

## Abstract

*Background and Objectives*: The risk of developing glycemic dysregulation up to overt diabetes mellitus (DM) after an episode of acute pancreatitis (AP) is increasingly being analyzed. We aimed to assess the changes in serum glucose levels associated with the first episode of AP, as well as the impact of dysglycemia on outcomes such as the severity of inflammation, the length of hospitalization, mortality, and the persistence of hyperglycemia at follow-up. *Materials and Methods*: All patients experiencing their first episode of AP, who presented to the Emergency Room (ER) between 1 January 2020 and 31 December 2023, were retrospectively included. On-admission serum glucose and peak serum glucose during hospitalization were the biological markers used to assess glucose metabolism impairment, and they were correlated with outcomes of AP. *Results*: Our study included 240 patients, 46.67% (112 patients) having a biliary etiology for an AP flare. Patients with COVID-19-associated AP exhibited the highest on-admission and peak serum glucose levels (244.25 mg/dL and 305.5 mg/dL, respectively). A longer hospital stay was noted in patients with peak serum glucose levels of ≥100 mg/dL (9.49 days) compared to normoglycemic patients (6.53 days). Both on-admission and peak glucose levels were associated with elevated CRP levels during hospitalization. A total of 83.78% of patients who received antibiotics exhibited on-admission hyperglycemia, and 72.07% had peak serum glucose levels of ≥100 mg/dL. The presence of hyperglycemia at follow-up was associated with both on-admission and peak serum glucose levels of ≥100 mg/dL, as well as with a longer stay, higher CRP levels, and antibiotic use during index admission. *Conclusions*: On-admission hyperglycemia predicts a higher inflammatory response in patients at the first episode of AP, while the presence of hyperglycemia during hospitalization is associated with imaging and biological severity and longer hospitalizations, indicating a more severe disease course. Both on-admission and peak in-hospital hyperglycemia were identified as risk factors for sustained hyperglycemia at follow-up.

## 1. Introduction

Acute pancreatitis (AP) is one of the most common pathologies seen in emergency Gastroenterology departments, with increasing incidence [1]. Although benign in nature, it bears a significant mortality rate in severe forms associated with organ failure and complications [2]. 

The interplay between the exocrine and endocrine pancreatic functions is well recognized, and β-cell metabolism is significantly impaired in diseases of the exocrine pancreas [3]. 

**Figure 1:**
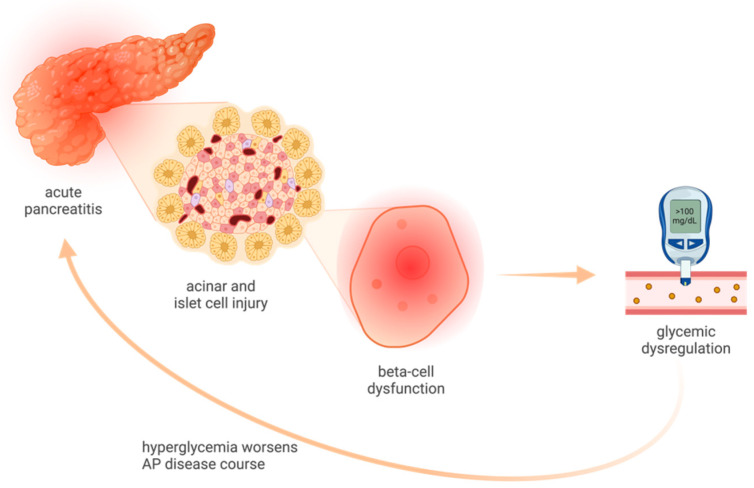
Connection between AP-related glycemia and disease course (created with BioRender.com, accessed on 10 April 2024).

Among predictors of outcomes in AP, on-admission glucose has been reported as an independent risk factor [4]. Also, hyperglycemia has been shown to be associated with the release of inflammatory cytokines, which can further contribute to a worsening of the disease course in AP [5] (Figure 1). As for other acute illnesses, stress hyperglycemia has been reported in AP [6], and there is a dose-dependent correlation with severity and mortality in AP [7]. Besides acting as an indicator for AP severity, glucose levels may also impact recovery from an AP flare [8]. Considering the significant impact of glucose dysregulation in AP outcomes, an important role stands for therapeutic interventions for glycemic control during an AP flare. 

There is a paucity of data regarding metabolic abnormalities at diagnosis and during an AP episode in Romanian patients. In this study, we aimed to analyze the glucose metabolism impairment in association with the first episode of AP, assessing correlations between on-admission and in-hospital glycemia and the AP outcomes, represented by the length of hospitalization and the mortality rate.

## 2. Materials and Methods

### 2.1. Study Design

This is a retrospective study that included patients diagnosed with a first flare of AP, who presented to the Emergency Room (ER) of the Central Military Emergency University Hospital, Bucharest, from 1 January 2020 to 31 December 2023. AP diagnosis was set according to the 2013 IAP/APA guideline based on two out of three established criteria: the presence of typical abdominal pain, an elevation of pancreatic enzymes over three times above the normal threshold, and characteristic imaging features of AP.

### 2.2. Participants

Patients eligible for our study were required to meet the following inclusion criteria: adults over the age of 18, experiencing their first episode of AP and having at least two recorded glucose values on venous samples. The first glucose level must have been measured either upon admission or within the first 24 h following ER presentation (referred to as ‘on-admission serum glucose level’) and the second measurement taken at any point during hospitalization. If multiple glucose readings were available during the hospital stay, the highest recorded level (referred to as ‘peak serum glucose’) was used for analysis. Exclusion criteria for this study included the following: a previous history of AP, imaging evidence of chronic pancreatitis, and incomplete data regarding both the on-admission glycemic level and the peak serum glucose level during hospitalization. The patient search was conducted using ICD-10 coding, with AP being the main diagnosis at discharge. 

### 2.3. Variables

On-admission serum glucose levels were assessed using venous blood samples as part of the blood work conducted upon ER presentation. For patients lacking initial glycemic data, the glucose level recorded within the first 24 h after admission was considered the on-admission serum glycemia. It is important to note that these measurements cannot be classified as fasting glycemia, since they are taken upon ER presentation. However, it is pertinent to mention that many of these patients may not have had oral food intake for prolonged hours before presentation due to intense abdominal pain, nausea, vomiting, and a lack of appetite.

The peak serum glucose was defined as the highest value recorded among all serum glucose measurements during the hospital stay.

The glycemia at follow-up refers to the fasting venous serum glucose measured during a scheduled re-evaluation after the AP flare. On-admission, during hospitalization, and at follow-up serum glucose levels were performed on venous blood samples, using the AU5822 clinical chemistry analyzer (Beckman Coulter, Brea, CA, USA). 

Normal serum glucose levels were identified as values less than 100 mg/dL, while hyperglycemia was defined as a serum glycemia of 100 mg/dL or higher.

Moreover, in a subgroup of patients, glycosylated hemoglobin (HbA1c) was performed on venous blood samples, using the Sebia Capillary analyzer (Paris, France). Patients were divided into 3 groups according to HbA1c levels, using the thresholds recommended by the American Diabetes Association: below 5.7%, between 5.7% and 6.4%, and higher or equal to 6.5% [9].

Serum C-reactive protein (CRP) is used to assess inflammation during hospitalization for AP. The determination of CRP levels was performed on venous blood samples, using the AU5822 clinical chemistry analyzer (Beckman Coulter, Brea, CA, USA). Peak CRP was the highest value of CRP obtained during the entire hospital stay, and its level was regarded as the biological marker of severity of AP, while imaging severity was evaluated using the Balthazar score on computed tomography (CT) scans. In instances of AP with mild clinical and biological severity, along with a satisfactory visualization of the pancreas on an abdominal ultrasound, a CT scan was not carried out at the decision of the clinician, and the Balthazar score was not documented.

The need for antibiotic therapy was assessed as a binary value, as our aim was only to note if the patients did or did not require antibiotics during hospitalization. The type or the class of the antibiotics was not retained.

### 2.4. Statistical Analysis

We used the Chi-squared test to assess whether there is a significant association between two categorical variables, with subsequent evaluation using the associated *p* value. The T-test was employed to analyze differences in the mean age between groups classified based on the presence or absence of hyperglycemia. The Pearson correlation coefficient (r) was calculated to assess the relationships between the length of hospitalization, CRP levels, and serum glucose levels. The ANOVA test was applied to identify if there are statistically significant differences in the mean serum glucose levels (both on-admission and peak glycemia) across different Balthazar score groups. Figures were generated using the Microsoft Excel software (Version 16.77).

### 2.5. Ethics

Ethical approval was obtained from the ethical committee board of the “Dr Carol Davila” Central Military University Emergency Hospital (489/28.01.2022).

## 3. Results

Altogether, 240 patients were recruited for the purpose of this study, including 149 males (62%), with a mean age of 56.3 years. Patient demographics, along with disease characteristics, according to the on-admission glycemia and peak serum glycemia, are summarized in Table 1.

### 3.1. Etiology

**Figure 2:**
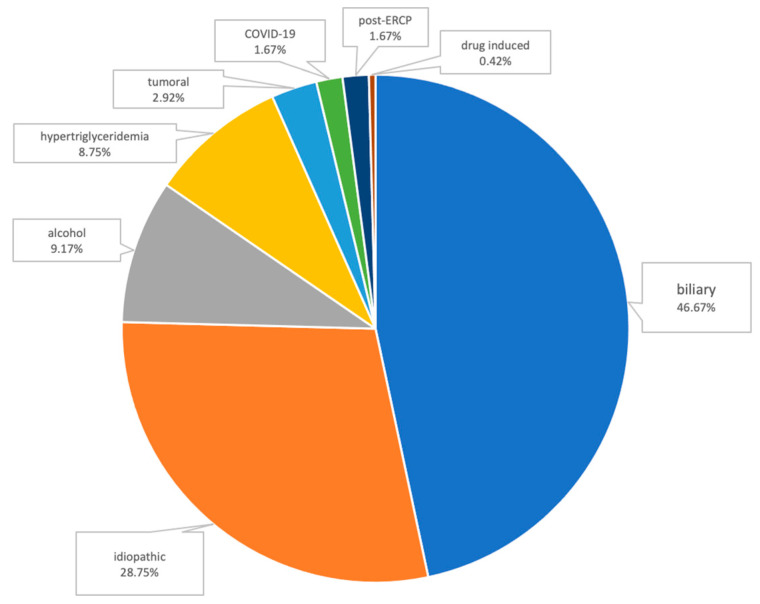
Etiologies of AP.

The most frequent etiologies of AP were the following: biliary AP (112 patients, 46.67%); alcoholic AP (22 patients, 9.17%); and hypertriglyceridemic AP (21 patients, 8.75%). Other causes were the following: pancreatic tumoral obstruction (7 patients), post-ERCP (4 patients), AP associated with SARS-CoV-2 infection (4 patients), and drug-induced AP (1 patient, AP associated with mesalazine use). In 69 cases (28.75%), due to the absence of a common identifiable cause after initial work-up, we classified these patients as having idiopathic AP (Figure 2). 

### 3.2. CT Imaging

**Figure 3:**
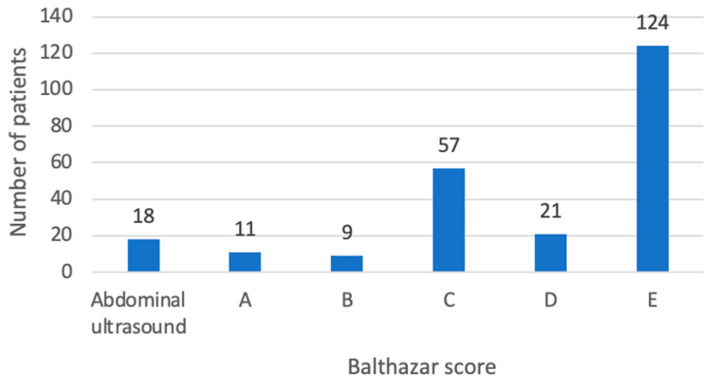
Distribution of patients according to Balthazar score and abdominal ultrasound.

In the study cohort, 18 out of 240 patients were assessed by abdominal ultrasounds only and did not require CT scans, due to a mild clinical and biological presentation. Consequently, the Balthazar score was not documented for these individuals. In the remaining 222 patients, Balthazar E comprised the most frequent group (124 patients), followed by the Balthazar C (57 patients) and D (21 patients) scores (Figure 3).

**Figure 4:**
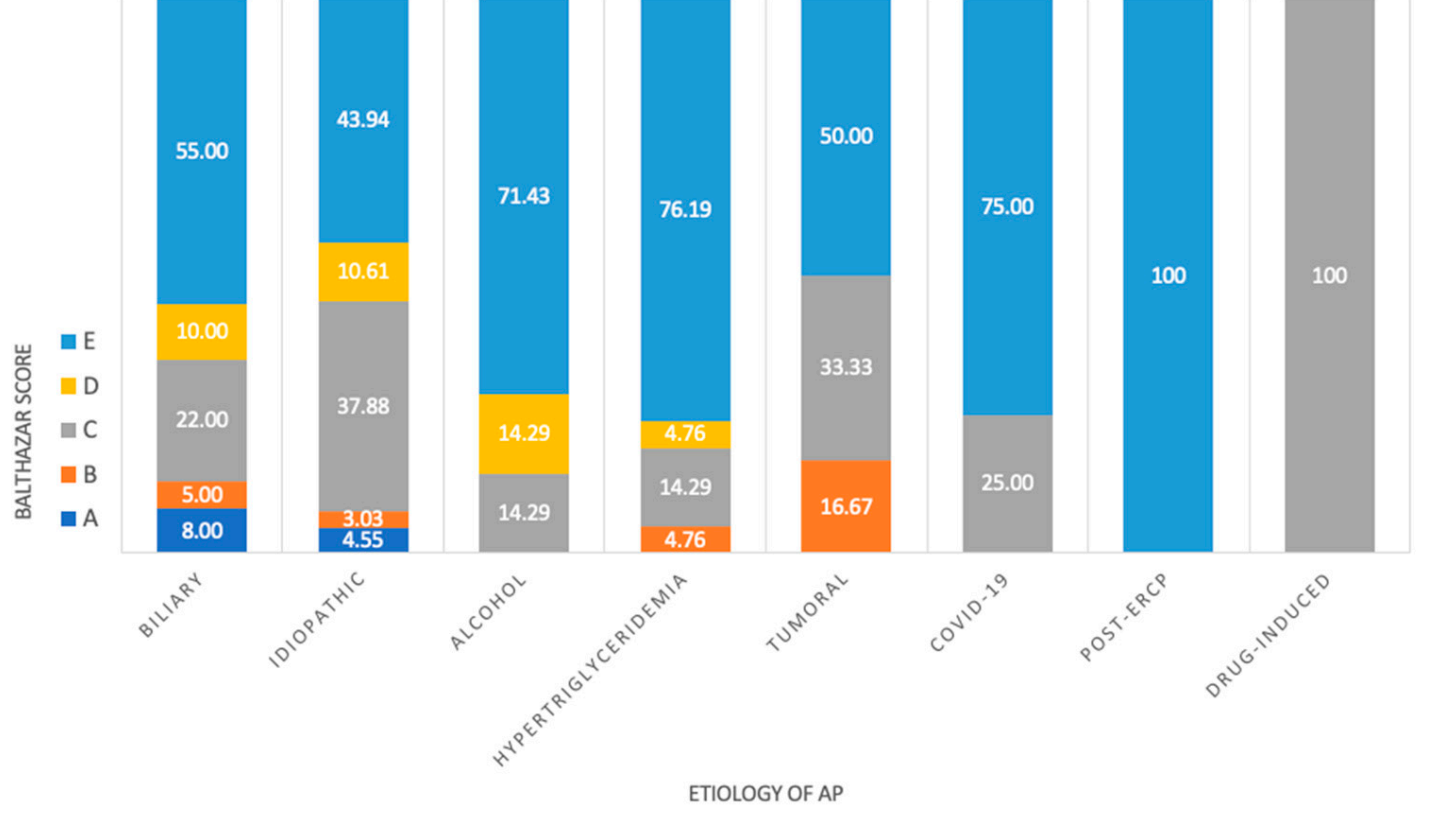
Cross-tabulation of etiology of AP and Balthazar score.

No statistically significant association between the etiology of AP and the Balthazar score was observed in our study (*p* value = 0.509) (Figure 4).

### 3.3. Serum Glucose Levels

Among the recruited patients, 198 (82.5%) exhibited hyperglycemia upon ER presentation. The average on-admission glycemia was measured at 150.97 mg/dL, with a range extending from 46 mg/dL to 571 mg/dL. Our analysis revealed no statistically significant differences for on-admission serum glucose levels across the various Balthazar score groups (*p* = 0.274). However, a significant variation was observed in peak serum glucose levels among the different Balthazar score groups, with patients in the Balthazar B and E groups having higher peak glucose levels (*p* = 0.021) (Table 2).

**Figure 5:**
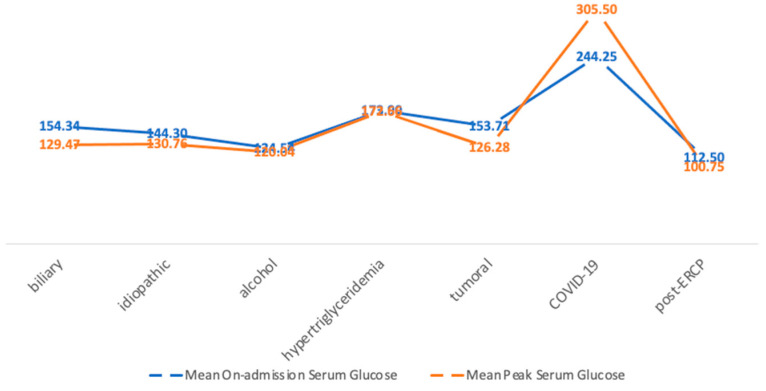
Mean on-admission and peak serum glucose levels in each etiology subgroup.

The highest mean on-admission glucose level (244.25 mg/dL) was observed in patients with COVID-19-associated AP, likely due to stress-induced hyperglycemia. Similarly, the highest peak serum glucose during hospitalization was also recorded in patients with COVID-19-associated AP (an average of 305.5 mg/dL) (Figure 5). However, the correlation between COVID-19 etiology and on-admission hyperglycemia was weak (r = 0.222). A weak correlation was also noted between the COVID-19 etiology and the peak glycemia during hospitalization ≥100 mg/dL (r = 0.327).

### 3.4. Pre-Existing DM

**Figure 6:**
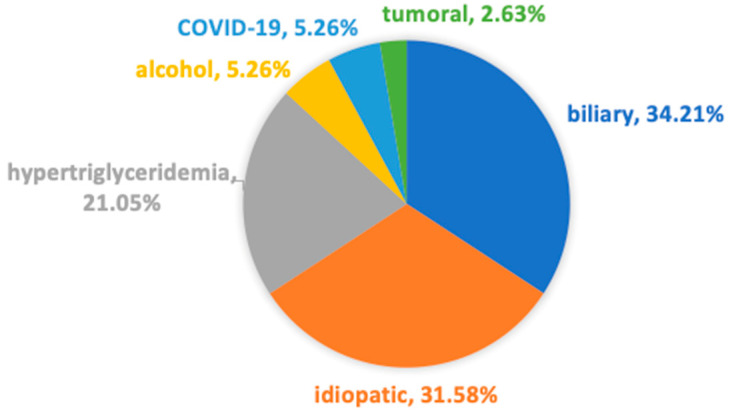
Etiology of AP in patients with pre-existing DM.

In our study, 38 patients (15.83%) had pre-existing type II DM. There was no patient with type I DM. The most common etiology of AP in patients with pre-existing DM was biliary (13 patients, 34.21%) and idiopathic (12 patients, 31.58%) (Figure 6).

### 3.5. The Length of Hospitalization

The average length of hospitalization was 8.47 days, extending to 8.79 days in patients with pre-existing DM.

Patients with on-admission normoglycemia experienced an average hospital stay of 8.62 days, while those with on-admission hyperglycemia had a slightly lower average stay of 8.44 days, indicating no significant relationship between the length of hospital stay and the serum glucose levels at the onset of an AP flare.

In contrast, a notable difference was observed in relation to peak serum glucose levels during hospitalization. Patients with a peak serum glucose level below 100 mg/dL had an average stay of 6.53 days, while those with levels equal to or greater than 100 mg/dL had longer hospital stays, averaging 9.49 days. Consequently, a weak positive correlation was identified between the peak serum glucose level and the length of hospitalization (*p* value < 0.01). 

### 3.6. The CRP Levels

In our analysis, we observed the peak CRP levels recorded during hospitalization, with an average value of 182.89 mg/L. A positive correlation between the peak CRP level and on-admission glycemia was seen (*p* < 0.05), indicating that higher initial serum glucose levels tend to be associated with elevated CRP values during hospitalization. Additionally, a statistically significant relationship was identified between the peak CRP level and the peak serum glucose level during hospitalization (*p* = 0.0041), further supporting the link between increased glucose levels and inflammatory markers in acute settings (Table 1).

### 3.7. Antibiotic Therapy

In our study, 111 out of 240 patients (46.25%) were prescribed antibiotic therapy during hospitalization. Among those who received antibiotics, 93 (83.78%) had on-admission hyperglycemia, and 80 (72.07%) had a peak serum glucose of ≥100 mg/dL. There was no difference in antibiotic requirement in patients with pre-existing DM (17/38, 44.73%) compared to non-diabetics (46.53%).

### 3.8. Mortality

The in-hospital mortality rate in our study was 5% (12/240), including 4 females and 8 males with a mean age of 70.42 years. The predominant etiology of AP in deceased patients was biliary (50%, 6 patients), followed by idiopathic causes (25%, 3 patients), tumoral causes (16.67%, 2 patients), and COVID-19 infections (8.33%, 1 patient). Only two of the deceased had pre-existing DM.

We observed that patients with on-admission normoglycemia had a higher mortality rate of 9.5% compared to those with on-admission hyperglycemia who had a mortality rate of 4%. On the other hand, peak serum glucose levels of ≥100 mg/dL were present in the majority of deceased patients, with a mortality rate of 5.7% compared to 3.6% among those with peak serum glucose levels of < 100 mg/dL. Thus, the mortality was associated with a peak serum glucose of ≥100 mg/dL rather than with on-admission hyperglycemia (r = 0.209, *p* value = 0.0085).

### 3.9. HbA1c

After excluding patients with pre-existing DM, a subgroup analysis evaluating the level of HbA1c was performed on 54 patients, 28 of them having an HbA1c of <5.7%, 22 having values ranging between 5.7% and 6.4%, and 4 patients having an HbA1c of ≥6.5%. Both on-admission serum glucose levels and peak serum glycemia were higher in patients with increased HbA1c levels. Patients with an HbA1c greater than 6.5% had also higher CRP values, longer hospitalizations, and a greater need for antibiotics (Table 3). 

### 3.10. Follow-Up

**Figure 7:**
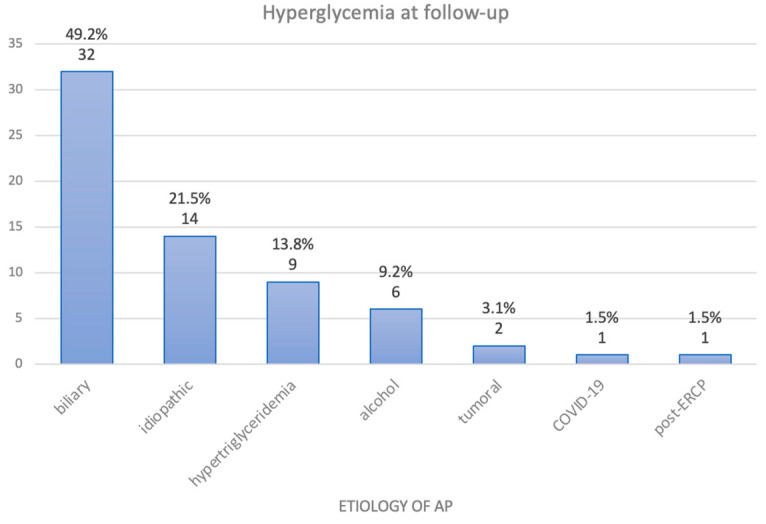
Distribution of patients with hyperglycemia at follow-up, categorized by the etiology of AP.

Out of the 240 patients included in this study, 140 (58.33%) had a follow-up in the first 6 months after the AP flare. A total of 65 out of these 140 patients (46.42%) had hyperglycemia at the re-evaluation, of whom 21 were previously diagnosed with DM. The mean age of patients experiencing persistent hyperglycemia during follow-up was 57.64 years, with 61.54% (40 patients) being male. Looking in retrospect, among those with hyperglycemia during follow-up, 60 patients (92.31%) had on-admission hyperglycemia and 80% (52 patients) had peak serum glucose levels of ≥100 mg/dL. Patients with hyperglycemia at follow-up had an average hospital stay of 9.97 days. We observed a higher peak CRP level during initial hospitalization in patients with hyperglycemia at follow-up, at an average of 200.67 mg/dL. Furthermore, 31 of these 65 patients (47.7%) received antibiotic therapy during their initial hospitalization for AP. Concerning the etiology of AP in patients with hyperglycemia at follow-up, 49.2% of the patients (32 patients) had biliary AP, 21.5% (14 patients) had idiopathic AP, 13.8% (9 patients) had hypertriglyceridemic AP, 6 patients (9.2%) had alcohol-induced AP, 2 patients (3.1%) had tumoral causes of AP, 1 patient (1.5%) had COVID-19-associated AP, and 1 patient (1.5%) had post-ERCP AP (Figure 7).

## 4. Discussion

In the current paper, we analyzed glycemic dysregulation patterns in AP, a connection born both as a consequence of an acute illness with oxidative stress and as a relationship between the endocrine and exocrine pancreas. 

The metabolic stress induced by inflammation is responsible for hyperglycemia as an adaptive response in order to protect the vital organs. Hyperglycemia is often noted in ICU patients as a response to cortisol release in stress conditions, as well as the presence of excessive counterregulatory hormones (glucagon, growth hormone, catecholamine) and high levels of cytokines (tumor necrosis factor α and interleukin-1). Altogether, these lead to an increased hepatic gluconeogenesis and an impaired insulin-mediated glucose uptake into skeletal muscles [10]. Conversely, hyperglycemia has deleterious effects, increasing the severity of cellular damage by inducing an intracellular glycose overload and acute glucotoxicity. This condition promotes oxidative stress, inflammation, endothelial dysfunction, coagulation, and osmotic diuresis while inhibiting vasodilatation, thereby creating conditions for ischemic injuries. As all pancreatic cells are damaged in AP, the diminished insulin secretion by pancreatic β cells further intensifies hyperglycemia, perpetuating a vicious cycle [6].

In our study, 82.16% of patients had hyperglycemia upon hospital admission. We observed that patients with on-admission hyperglycemia were furthermore susceptible to higher levels of inflammation during hospitalization, as indicated by the peak CRP levels (a mean peak CRP of 195.25 mg/dL). Interestingly, patients with pre-existing DM also displayed comparable peak CRP levels, with a mean peak CRP of 177.32 mg/L, in the context of the known proinflammatory state of patients with DM. However, despite the elevated CRP levels in patients with on-admission hyperglycemia, our findings did not reveal a significant increase in the length of hospital stay for these patients. Instead, the duration of hospitalization appeared to be more closely related to the presence of hyperglycemia throughout the hospitalization period.

Nearly half of the patients included in this study received antibiotic treatment. Among them, 83.78% had on-admission hyperglycemia, and around 72% had a peak serum glucose of ≥100 mg/dL during hospitalization. These findings suggest that hyperglycemia may be linked to an increased risk of infection, thereby necessitating more frequent antibiotic use. Additionally, 17 out of the 38 patients (44.7%) with pre-existing DM required antibiotic therapy.

Regarding the mortality after the first episode of AP, in our study, it was associated, albeit weakly, with a peak serum glucose of ≥100 mg/dL rather than with on-admission hyperglycemia. Similarly, Egi et al. demonstrated that hyperglycemia during hospitalization in an intensive care unit for an episode of acute pancreatitis is rather a negative prognostic feature for patients without pre-existing DM than for those with DM [11]. In our study, only two of the patients who died were previously diagnosed with diabetes, suggesting that the hyperglycemia observed in non-survivors was not due to pre-existing conditions but rather was associated with the acute phase of the disease.

Patients with DM are at a greater risk of developing AP, especially those with uncontrolled or untreated DM [12]. Moreover, pre-existing DM may be encountered in approximately 25% of AP patients [13]. 

In our study, 38 patients (15.7%) had a known diagnosis of type II DM, a rate significantly higher than the general prevalence of DM in Romania, which stands at 3.9% [14]. 

DM that develops after an episode of AP is commonly classified as type 3c DM (T3cDM), also known as diabetes of the exocrine pancreas. T3cDM is an emerging concept with growing prevalence, estimated to constitute between 5% and 10% of all diabetes cases in the Western population [15]. It is often misdiagnosed as T2DM because of the lack of antibodies that differentiate it from T1DM. However, a 2017 English study showed that its prevalence could be higher than the prevalence of T1DM [16]. As T3cDM affects all cells in the islet of Langerhans, it impairs the secretion not only of the insulin but also of glucagon, pancreatic polypeptide, incretin, and adipokines, leading to deleterious glycemic control and both insulin resistance and insulin deficiency [17].

Hyperglycemia is often considered a transient phenomenon, with most patients passing to normal glycemic levels after an acute episode of the illness. A 7-year Australian study on ICU patients revealed that 17% of the 17.074 non-diabetic patients had hyperglycemia, but only 4.8% developed T2DM following the acute illness. However, patients with stress-induced hyperglycemia were at a double risk of developing DM compared to those without hyperglycemia [18].

In our study, only 58.33% of the patients presented for follow-up, and nearly half of them (46.42%) exhibited hyperglycemia. We observed that on-admission hyperglycemia as well as a peak serum glucose of ≥100 mg/dL were risk factors for hyperglycemia at follow-up, as a significant majority of these patients (60 out of 65 patients, 92.31%) also had on-admission hyperglycemia, and 80% (52 patients) had a peak glycemia of ≥100 mg/dL during hospitalization. Additionally, patients with persistent hyperglycemia at follow-up also showed higher levels of CRP during their initial hospitalization, as well as a longer hospital stay, averaging 9.97 days.

However, a meta-analysis by Das et al. revealed no correlation between the severity of AP or the etiology and the risk of developing prediabetes or DM [19]. So, even if islet cell loss seems to be the easiest-to-understand mechanism of T3cDM after AP, it is probably not the only mechanism to be taken in consideration [20]. Other mechanisms have also been reported, such as autoimmunity, the presence of risk factors such as obesity, hypertriglyceridemia, dyslipidemia, chronic kidney disease, liver disease, local and systemic inflammation, and genetic involvement [20,21]. 

When looking at the link between DM and AP, we found out that the incidence of DM seems to increase in time after the episode of AP. A prospective longitudinal cohort study on AP patients described an increasing cumulative incidence of new-onset prediabetes and DM in time, with 20% at 6 months and 43% at 24 months [22]. Similarly, a meta-analysis by Das et al. [19] including 24 prospective clinical studies and 1102 patients at the first episode of AP found out that within 1 year after the first episode of AP, 15% of the patients developed newly diagnosed DM, with the risk increasing significantly at 5 years—relative risk 2.7 (95% CI 1.9 to 3.8) [19]. The increasing risk over time of developing DM after AP was also sustained in a meta-analysis including 31 studies and 13,894 subjects, revealing a pooled incidence of DM within 5 years after AP at 20%, going up to 37% in patients who were followed-up for more than 5 years [23]. However, the above-mentioned risk of developing DM has to be differentiated by transitory hyperglycemia and temporarily increased insulin resistance. In a study from Taiwan that included 2966 first-attack AP patients, the incidence of DM was 60.8% within 3 months, decreasing to 22.5% in patients that had a follow-up for more than 3 months [24].

HbA1c is a useful tool in clinical practice, mainly in the diagnosis and the follow-up of patients with DM. It indicates an average of serum glucose levels over the past 90 days [25]. The subgroup analysis in our study regarding the HbA1c level shows that patients with AP and higher levels of HbA1c had worse outcomes, in terms of CRP, hospital stay, or the need for antibiotics. Actually, patients with a higher HbA1c also had greater values of on-admission serum glucose levels and peak serum glucose levels. These data suggest that these might be patients with undiagnosed glycemic perturbations, and these patients could be at risk of a worse evolution of AP.

Regarding the correlation between the etiology of AP and glycemic disturbances, in our study, we found that patients with COVID-19 infections had higher levels of serum glucose both on admission and during hospitalization (peak serum glucose ≥ 100 mg/dL). However, the correlation between the COVID-19 etiology and serum glucose levels is slightly higher for a peak serum glycemia of ≥100 mg/dL, rather than for on-admission hyperglycemia. COVID-19-associated AP represents a newly recognized entity, with pathophysiology and long-term risks which are not yet fully understood. It is evident, however, that hyperglycemia as a result of a stress-induced release of a cascade of hormones that promote gluconeogenesis, which inhibit the peripheral use of glucose and increase insulin resistance, is a primary mechanism under consideration. Other mechanisms have also been theorized: while some authors have reported an expression of angiotensin-converting enzyme 2 (ACE-2) as a mediator of GI involvement in COVID-19 infections [26,27], Schepis et al. described the presence of SARS-CoV-2 RNA in a pancreatic pseudocyst sample, raising the question if COVID-19-related AP could be a form of a viral pancreatitis, or if the inflammatory cells are transporters for the virus, or even if there is a retrograde contamination from the intestinal tube [28].

During the pandemic, it was noted that patients with DM are at a greater risk of a severe form of COVID-19 infection. On the other hand, even people without pre-existent DM were at a greater risk of hyperglycemia during COVID-19 infection, and many of them developed new-onset DM associated with COVID-19 infection [29]. A Chinese study reported that 20.8% of hospitalized patients for COVID-19 were newly diagnosed with DM and 28.4% with dysglycemia (fasting glucose 5.6–6.9 mmol/L and/or HbA1c 5.7–6.4%) [30]. Patients with COVID-19 and hyperglycemia at admission had a greater mortality and worse inflammatory profiles [31]. Additionally, those with newly diagnosed DM had a greater risk of admission in an intensive care unit, a greater risk of systemic complications, and a longer hospitalization [30].

Regarding pancreatitis arising post-ERCP, this represents a specific subtype of AP as a procedural complication after instrumentation of the papilla. Despite routinely implemented preventive strategies [32], ERCP inadvertently induces AP in a small proportion of patients, and of them, only a minority will evolve to severe forms. The pancreatic insult triggered by ERCP leads to a disruption of pancreatic endocrine function and as a consequence of β-cell damage or functional impairment. This, in turn, generates hyperglycemia, which can be further exacerbated by systemic stress responses in severe forms of AP. Interestingly, the mechanism behind glucose dysregulation in patients with post-ERCP AP is not only related to β-cell injury but also to the procedure itself, by means of a metabolic response to the ERCP maneuver with insulin resistance, which is independent of AP occurrence [33]. Moreover, contrasting the negative impact of DM on AP outcomes, some authors have found the presence of DM to have a protective effect on the rate of post-ERCP pancreatitis [34,35]. Not least, a potential confounder in serum glucose measurements might be represented by glucagon administration during ERCP for the purpose of reducing excessive duodenal motility, which could interfere with cannulation. In our study, none of the patients with post-ERCP pancreatitis received periprocedural intravenous glucagon.

In terms of glycemic fluctuations and imagistic features of acute pancreatitis (AP), our study revealed no statistically significant differences in on-admission glycemia across the various Balthazar score groups. However, we observed a statistically significant variation in peak serum glucose levels among the different Balthazar score groups (*p* = 0.021).

No differences in CT findings between patients with DM and those without DM were noted by Kikuta et al. [36]. Similarly, the severity of AP had only a minor effect on the incidence of prediabetes or newly diagnosed DM, as shown in a meta-analysis focusing on newly diagnosed DM following AP [19]. On the other hand, some authors suggested that patients with severe AP are at a risk of developing DM, with this risk being correlated to the extent of necrosis [37]. A study by Garip et al. on 109 patients with AP found that 56.4% of patients with severe AP were reported with endocrine dysfunction compared with 23.2% of those with mild AP [38]. Moreover, necrotizing AP has been reported to have a higher incidence of AP-induced DM in comparison to subjects without necrosis [23]. The same study by Garip et al. mentioned that all the patients that needed necrosectomy developed endocrine dysfunction [38]. A systematic review and meta-analysis on risk factors for developing DM after AP found a significant association between the existence of pancreatic necrosis and the development of prediabetes or DM, with significantly higher odds of developing DM in patients with a necrosis of over 50% of the pancreas [21].

### Limitations

Our work has several limitations. Firstly, it is important to highlight that our study is a retrospective study and that part of the enrollment period overlapped with the COVID-19 pandemic. The period of pandemic was characterized by restricted hospital access, primarily reserved for emergency cases, while patients with lower severity and non-emergency conditions were encouraged to seek home treatment and consultations with their general practitioners. Consequently, the data presented in this study may be influenced, particularly in terms of the etiology and severity of AP. For patients who developed AP post-ERCP, we included their on-admission serum glucose levels to maintain consistency within this study’s framework. However, it is important to note that these patients did not have AP upon admission; rather, AP developed iatrogenically during their hospital stay. Another limitation of our study is its focus solely on serum glucose level changes. It would have been significantly informative to also examine variations in HbA1c levels both at admission and during the follow-up period for all the patients included in this study. Our study could have benefitted from a correlation of the obtained results with the patients’ body mass indexes (BMIs), but data regarding BMIs were not available in the medical records of all patients.

## 5. Conclusions

Our study identified that hyperglycemia at admission is linked to higher levels of inflammation and an increased requirement for antibiotic treatment during the initial episode of AP. More importantly, hyperglycemia during hospitalization, indicated by peak serum glucose levels over 100 mg/dL, is a risk factor for prolonged hospital stays and increased mortality. Furthermore, both an on-admission hyperglycemia and a peak serum glycemia of over 100 mg/dL were identified as risk factors for sustained hyperglycemia at follow-up. Consequently, they indicate a heightened risk for the development of diabetes mellitus following an episode of AP.

## Figures and Tables

**Table 1:** Baseline characteristics of the patients, the disease, and the outcomes according to on-admission glycemia and peak serum glycemia.

	On-Admission Glycemia			Peak Serum Glycemia		
	<100 mg/dL	≥100 mg/dL	*p*-value	<100 mg/dL	≥100 mg/dL	*p*-value
**n**	42	198		83	157	
**Age (years), mean**	49.36	57.36	0.0012	53.35	57.97	0.029
**Male, n (%)**	21 (50)	128 (64.6)	0.1092	39 (47)	110 (70)	0.00077
**Pre-existing DM, n (%)**	1 (2.4)	37 (18.7)	0.0165	3 (3.6)	35 (22.3)	0.00034
**Balthazar score, n**						
A	5	6	0.036	5	6	0.652
B	0	9	0.336	2	7	0.662
C	13	44	0.313	24	33	0.227
D	1	20	0.191	12	9	0.042
E	20	104	0.683	36	88	0.083
**Etiology, n**						
Biliary	17	95	0.475	41	71	0.631
Idiopathic	14	55	0.593	26	43	0.623
Alcohol	2	20	0.427	3	19	0.053
Hypertriglyceridemia	6	15	0.273	6	15	0.714
Tumoral	0	7	0.464	3	4	0.949
COVID-19	1	3	0.730	0	4	0.349
Post-ERCP *	1	3	1.0	3	1	0.237
Drug-induced	1	0	0.391	1	0	0.745
**Length of hospitalization (days), mean**	8.62	8.44	0.8755	6.53	9.49	0.00105
**CRP, mean (mg/dL)**	124.64	195.25	0.0017	150.92	199.80	0.0041
**Antibiotherapy, n (%)**	18 (42.9)	93 (47)	0.7526	31 (37.3)	80 (51)	0.0608
**Mortality, n (%)**	4 (9.5)	8 (4)	0.2752	3 (3.6)	9 (5.7)	0.6857

Abbreviations: * ERCP, endoscopic retrograde colangiopancreatography.

**Table 2:** Mean on-admission and peak glycemia in each Balthazar score group ± standard deviation, SD.

Balthazar Score	Mean On-Admission Serum Glucose (mg/dL)	Mean Peak Serum Glucose (mg/dL)
A	107.54 ± 28.79	101 ± 27.14
B	182.66 ± 107.72	174.66 ± 87.90
C	146.68 ± 82.14	120.82 ± 52.49
D	138.19 ± 62.18	114 ± 46.93
E	157.68 ± 84.35	145.55 ± 77.92

**Table 3:** HbA1c subgroup—characteristics and outcomes.

HbA1c	<5.7%	5.7–6.4%	≥6.5%
**Number of patients (%)**	28 (51.9)	22 (40.7)	4 (7.4)
**On-admission glycemia (mean ± SD)**	119.21 ± 23.25	148 ± 46.13	273.5 ± 87.37
**Peak serum glycemia (mean ± SD)**	109.62 ± 29.62	122.77 ± 25.09	259 ± 105.66
**CRP (mean)**	170.29	249.99	294.53
**Length of hospitalization (mean)**	8.03	12.95	26.25
**Need for AB (%)**	60.71	68.18	75

## Data Availability

Datasets are available from the corresponding author.
